# The gut microbiota-SCFA-inflammation axis in patients with AECOPD

**DOI:** 10.1371/journal.pone.0312606

**Published:** 2025-01-09

**Authors:** Hengjing Zhu, Chen Wu, Haiyan Wu, Juan Liu, Wu Ye, Tian Zhao, Zhijun Li

**Affiliations:** 1 Zhejiang Chinese Medical University, Hangzhou, Zhejiang Province, China; 2 Department of Respiratory Medicine in Jiashan County Second People’s Hospital, Jiaxing, Zhejiang Province, China; 3 Department of Respiratory Medicine in Zhejiang Hospital, Hangzhou, Zhejiang Province, China; Charotar Institute of Applied Sciences: P D Patel Institute of Applied Sciences, INDIA

## Abstract

**Objectives:**

The aim of the study was to explore the alteration of microbiota and SCFA in gut and inflammation in acute exacerbation chronic obstructive pulmonary disease (AECOPD) patients, and to test the hypothesis that a disorder of gut microbiota will lead to the alteration of SCFA, which will aggravate inflammation in AECOPD patients.

**Methods and results:**

24 patients with AECOPD and 18 healthy volunteers were included in the study. Gut microbiota were analyzed by 16S rDNA and serum was used to detect levels of inflammatory factors by ELISA. Fatty acid concentrations were determined in lumen via gas chromatography-mass spectrometry. The richness and diversity of gut microbiota were decreased in AECOPD patients. β-diversity analysis revealed differences between AECOPD patients and healthy controls. *p*_*Bacteroidetes*, *g_Paraprevotella*, *g_Ruminococcus2*, *g_Parasutterella*, *o_Rhodospirillales*, and *g_Romboutsia* in the healthy controls and *p_Firmicutes*, *o_Actinomycetales*, *f_Actinomycetadeae*, *g_Actinomyces*, *g_Mogibacterium*, *f_Veillonellaceae*, *f_Enterococcaceae*, and *g_Enterococcus* in AECOPD patients were the most abundant microbiota. SCFA levels were decreased in patients with AECOPD. In addition, the results demonstrated that except for a reduction in IL-6, there was no change in inflammatory markers in AECOPD patients.

**Conclusion:**

In AECOPD patients, the gut microbiota-SCFA-inflammation axis is augmented, with decreased diversity and abundance of gut microbiota, leading to a reduction in SCFA and an imbalance of inflammation.

## Introduction

Chronic obstructive pulmonary disease (COPD) is an inflammatory lung disorder with characteristics of airflow limitation, lung function impairment, and with several clinical presentations, such as cough, expectoration, and dyspnea. The incidence of COPD has increased worldwide, and it will become the third most prevalent cause of death by 2030 [1]. According to the results of the Pulmonary Health Observational Study in China, there are nearly 100 million COPD patients in the country [2]. Some COPD patients experience frequent exacerbations (episodic worsening of COPD symptoms) which results in increasing morbidity and mortality.

COPD is accompanied by changes in the structure of the intestinal microbiome [3]. The gut microbiota-short chain fatty acid (SCFA)-inflammation axis indicates that gut microbiome alterations can aggravate or alleviate lung inflammation by affecting the production of SCFA. Healthy gut microbiota contains a variety of intestinal microorganisms, and gut microbiota imbalance is closely related to respiratory diseases. [4–6]. A study by Li *et al*. showed that COPD patients had a remarkable reduction of gut microbial diversity and composition compared with a healthy population [7]. Sun *et al*. also found dynamic changes of the gut and lung microorganisms during acute episodes of COPD [8]. In addition, other published findings show that there are changes in the abundance and composition of the fecal microbiome between stable COPD and AECOPD patients, with a reduction of diversity and richness of taxonomic composition of fecal microbiota, which suggests that variations in fecal microbiota may be associated with COPD progression [9]. However, the effect of the change of gut microbiota on the SCFA produced in the gut still needs to be explored in AECOPD patients.

The main source of SCFA is production gut microflora, which contributes to maintaining the balance of pro-inflammatory and anti-inflammatory markers by regulating the size and function of Treg cells [10]. Mao *et al*. found that changes to the composition of gut microbiota, leads to the reduction of acetate, butyrate, and propionate levels and weaken the mucosal immunity function in COPD rats [11]. Another study indicated that the probiotic administration of *Bifidobacterium longum* subsp. *longum* reduced lung inflammation, inflammatory cytokine and adhesion factor expression, and alleviated cigarette smoke-induced depletion of cecum butyrate [12]. Furthermore, several studies reported that the potential therapeutic value for gut microbiota and metabolites from a high-fiber diet inhibited local and systemic inflammation in a rat emphysema model [13].

Local and systemic inflammation is frequent in patients with COPD, it has been reported that the inflammation and symptoms are aggravated during acute exacerbation of chronic obstructive pulmonary disease (AECOPD), and resolved after intervention [14]. Research from Chen et al. suggests that elevated levels of TNF-a and IL-10 in serum may be associated with COPD. Additionally, IFN-γ and IL-6 might be potential biomarkers for the further deterioration of lung disease patients [15]. Another research conducted by Obling *et al* suggested that patients with COPD and high upper airway symptoms displayed signs of eosinophilic and neutrophilic inflammation with elevated IL-1β in serum [16]. The alterations in the gut microbiota can contribute to the worsening of lung inflammation. Short-chain fatty acids (SCFAs), which are produced by the gut microflora, can alleviate lung allergic inflammation [17]. However, there is a lack of research to exploring the changes in the gut microbiota-SCFA-inflammation axis during the acute exacerbation period of COPD.

Looking back at previous studies, We identified gut microbiota using 16S rRNA sequencing and detecting the level of SCFA by using lumen via gas chromatography-mass spectrometry to test the hypothesis that a disorder of gut microbiota will lead to the alteration of SCFA, which will aggravate inflammation in AECOPD patients. This will help us better understand the connection between AECOPD and gut microbiota.

## Materials and methods

### Study population and diagnostic criteria

All individuals were recruited from Zhejiang Hospital or The First People’s Hospital of AnJi county, China, between January 2022 and May 2022. According to the diagnosis standards of AECOPD [18], spirometry was performed to determine the presence of COPD, and AECOPD was defined as the period when clinical symptoms, such as dyspnea, cough, and expectoration, were aggravated. Based on the World Medical Association Declaration of Helsinki of 1975, as revised in 1983, this study was approved by the Ethics Committee of Zhejiang Hospital and The First People’s Hospital of AnJi county. Written informed consent was attained from all subjects. Adverse events that occurred during the study were monitored and recorded.

#### The exclusion criteria were as follows

1) patients who had taken probiotics, antibiotics, or immunosuppressants within the previous 2 weeks; 2) diagnosis of other lung disorders, such as community-acquired pneumonia, upper respiratory tract infection, acute bronchitis, bronchiectasis with infection, asthma, and lung cancer; 3) gastrointestinal diseases, tumors, diabetes, liver or kidney dysfunction, connective tissue disease, or other inflammatory diseases that may affect the dysbiosis of gut microbiota and imbalance of inflammation.

### Blood collection and analysis

#### ELISA detection

The serum was extracted from venous blood at the morning 6:00, and next centrifugation at 3000 rpm for 15 min, and stored at -70°C until analysis. According to the manufacturer’s instruction, ELISA kits were used to detect the concentrations of serum transform growth factor (TGF)-β (Xuran Biological, EH6481M, Shanghai, China), interleukin (IL)-1β (Xuran Biological, EH6281M, Shanghai, China), IL-6 (Xuran Biological, EH6306M, Shanghai, China), and tumor necrosis factor (TNF)-ɑ (Xuran Biological, EH6513M, Shanghai, China). An automatic biochemical analyzer (DENLEY DRAGON Wellscan MK 3, Inc., Thermo Fisher Scientific, FIN) was used to measure the serum level of inflammatory factors. The serum triglycerides, total cholesterol, low-density lipoprotein, and high-density lipoprotein were detected by an automatic biochemical analyser (UniCel DxC 800 Synchron, Beckman Coulter, Inc., Indianapolis, IN, USA).

#### Fecal bacterial DNA extraction

According to the manufacturer’s protocols, fecal bacterial DNA was extracted by The E.Z.N.A.^®^ DNA Stool Mini kit (Omega Bio-tek, Norcross, GA, U.S.). NanoDrop 2000 (Thermo Scientific) was used to detect DNA concentration and purity.

#### PCR amplification and 16S rDNA sequencing

The V3-V4 hypervariable region of bacterial 16S rDNA, which included 338F (5’-ACTCCTACGGGAGGCAGCAG-3’) and 806R (5’-GGACTACHVGGGTWTCTAAT-3’), was quantified by PCR amplification using GeneAmp PCR System 9700 (ABI Co., USA).) The PCR products were purified by the AxyPrep DNA Gel Extraction Kit (Axygen Biosciences, Union City, CA, USA), and then sequenced by Illumina MiSeq PE300 (Illumina, San Diego, CA, USA). The specific details of this process have been described in detail in a previous study [5, 6].

### Bioinformatics analysis of gut microbiota

The operational taxonomic unit (OTU) clustering of the sequences was determined based on 97% similarity. Species accumulation curve was used to reflect the sample size. Rank abundance distribution curve, Rarefaction and Shannon index were used to reflect the richness and evenness of species. Wilcoxon rank sum test was used to assess intergroup differences for the α diversity index. Principal component analysis (PCA) was applied for the β diversity analysis to evaluate the similarities and differences between different groups. Metastat was used to analyze the difference at different of levels between AECOPD and control group. Linear discriminant analysis (LDA) effect size (LEfSe) was used to estimate all differential bacteria between the two groups.

### Metabolite extraction and gas chromatography-mass spectrometry detection of fatty acids

Samples were collected in 2 mL eppendorf (EP) tubes and 1 mL H_2_O was added followed by mixing for 10 s. Next the sample was homogenized in a ball mill for 4 min at 40 Hz, then sonicated for 5 min, repeated 3 times while incubating in ice water. Samples were then centrifuged for 20 min at 5000 rpm, and the 0.8 mL of the resulting supernatant for each sample was transferred into an EP tube. 0.1 mL 50% H_2_SO_4_ and 0.8 mL of extracting solution were then added and the samples were mixed by vortex for 10 s, followed by shaking for 10 min and sonication for 10 min (incubated in ice water). After sonication, samples were centrifuged for 15 min (10,000 rpm, 4°C) and incubated at -20°C for 30 min. The supernatant was then transferred into a fresh 2 mL glass vial for gas chromatography-mass spectrometry (GC-MS) analysis.

SHIMADZU GC2030-QP2020 NX gas chromatography-mass spectrometer was used for GC-MS analysis. The system utilized a HP-FFAP capillary column. A 1 μL aliquot of the analyte was injected in split mode (5:1). Helium was used as the carrier gas, the front inlet purge flow was 3 mL/min, and the gas flow rate through the column was 1 mL/min. The initial temperature was kept at 80°C for 1 min, then raised to 200°C at a rate of 10°C/min for 5 min, then raised to 240°C at a rate of 40°C/min and maintained at that temperature for 1 min. The injection, transfer line, quad and ion source temperatures were 240°C, 240°C, 150°C, and 200°C. The energy was -70 eV in electron impact mode. The mass spectrometry data were acquired in Scan/SIM mode with the m/z range of 33–150 after a solvent delay of 3.5 min.

### Statistics analysis

Quantitative data are expressed as mean ± standard deviation (SD), t-test was used to analyze the differences among two groups. All statistical analyses were performed in R24 and GraphPad Prism 5.0 (GraphPad Software Inc., La Jolla, CA, USA). *P* values <0.05 was regarded as statistical significance.

## Results

### Basic clinical characteristics

For the present study, 24 patients with AECOPD and 18 healthy volunteers were recruited. Several parameters, such as age, pulmonary function, blood gas analysis, and the results of urine and blood tests were analyzed and are summarized in Table 1. The average age was 71.08 ± 6.52 years in the healthy control group and 61.00 ± 7.00 years in AECOPD patients (*P* = 0.00, healthy *vs*. AECOPD). As patients in the AECOPD group were in an acute episode of COPD, several parameters, such as white blood cells, neutrophils, and C-reactive protein were elevated in the AECOPD group compared to the healthy volunteers (*P*<0.05). Serum albumin and triglycerides were reduced in AECOPD patients (*P* = 0.00 and *P* = 0.015, respectively), which suggested that patients with AECOPD experienced excessive nutrients consumption. In addition, no other differences were identified between the AECOPD group and healthy group (all *P*>0.05).

**Table 1:** The basic clinical characteristics of subjects.

Variables	Control (n = 18)	AECOPD (n = 24)	*P*
Age (year)	71.08±6.52	61.00±7.00	<0.01
Height (CM)	151.11±38.59	151.11±38.59	0.332
Weight (Kg)	53.93±22.64	59.08±10.67	0.089
FEV1 (L)	3.12±1.14	2.07±0.67	<0.01
FEV1/FVC (%)	82.33±11.41	51.67±9.29	<0.01
Albumin (g/L)	42.49±4.47	36.56±3.85	0.046
GPT (U/L)	23.06±9.38	18.54±19.02	0.361
GOT (U/L)	22.5±12.00	27.29±35.65	0.588
Creatinine (μmol/L)	63.83±11.53	55.59±22.38	0.130
TC (mmol/L)	4.37±2.00	3.97±0.85	0.439
TG (mmol/L)	1.32±0.73	0.85±0.22	0.015
LDL (mmol/L)	2.39±1.53	1.96±0.69	0.276
WBC (*10^9^/L)	5.45±1.80	8.55±4.08	0.002
NE (%)	61.88±11.51	72.91±18.28	0.030
CRP (mg/L)	3.16±4.35	55.42±55.45	<0.01

**NOTE** *CRP* C creative protein. *GOT* Glutamic oxaloacetic transaminase. *GPT* Glutamic pyruvic transaminase. *FEV1* Forced expiratory volume in 1 second. *FVC* Forced vital capacity. *NE* Neutrophil. *LDL* Low density lipoprotein. *TC* Total cholesterol. *TG* Triglyceride. *WBC* White blood cell.

### Diversity analysis of gut microbiota in AECOPD patients

**Fig 1:**
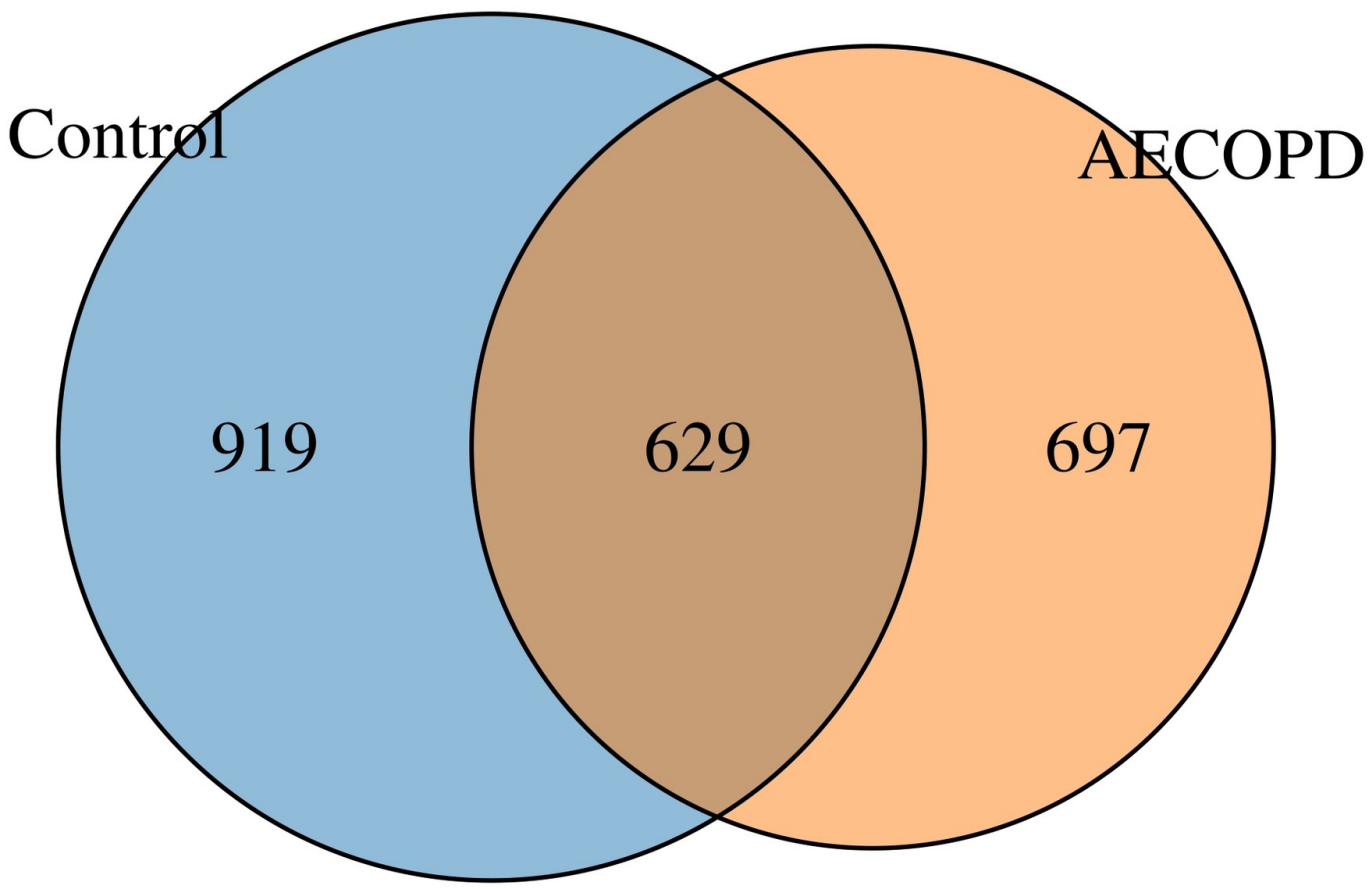
Venn diagram. The similarities and differences were analyzed in OTUs by a Venn diagram.

**Fig 2:**
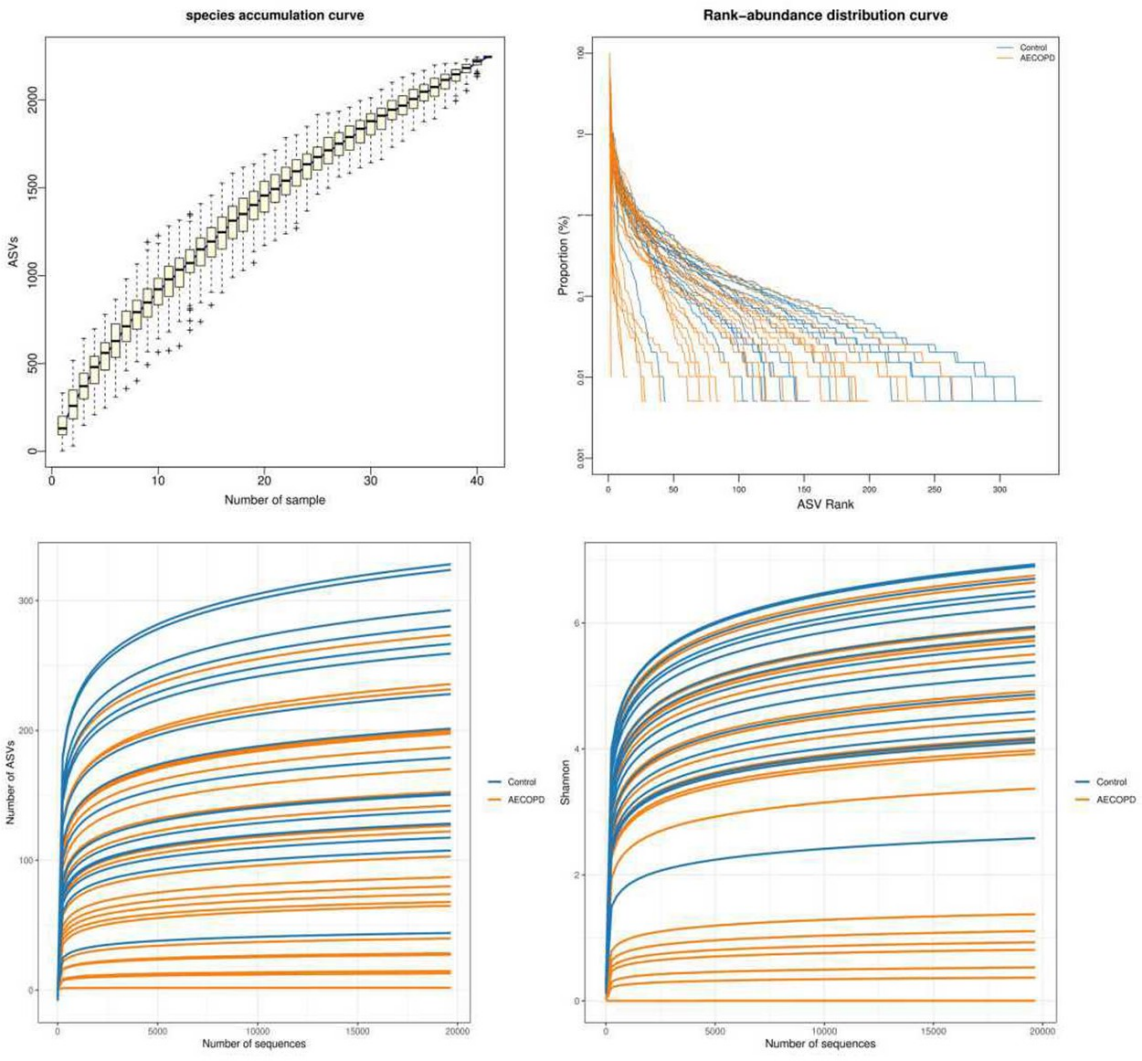
Alpha diversity analysis. The species accumulation curve, Rank-abundance distribution curve, rarefaction curve, and shannon index were used to reflect the species richness and diversity.

**Fig 3:**
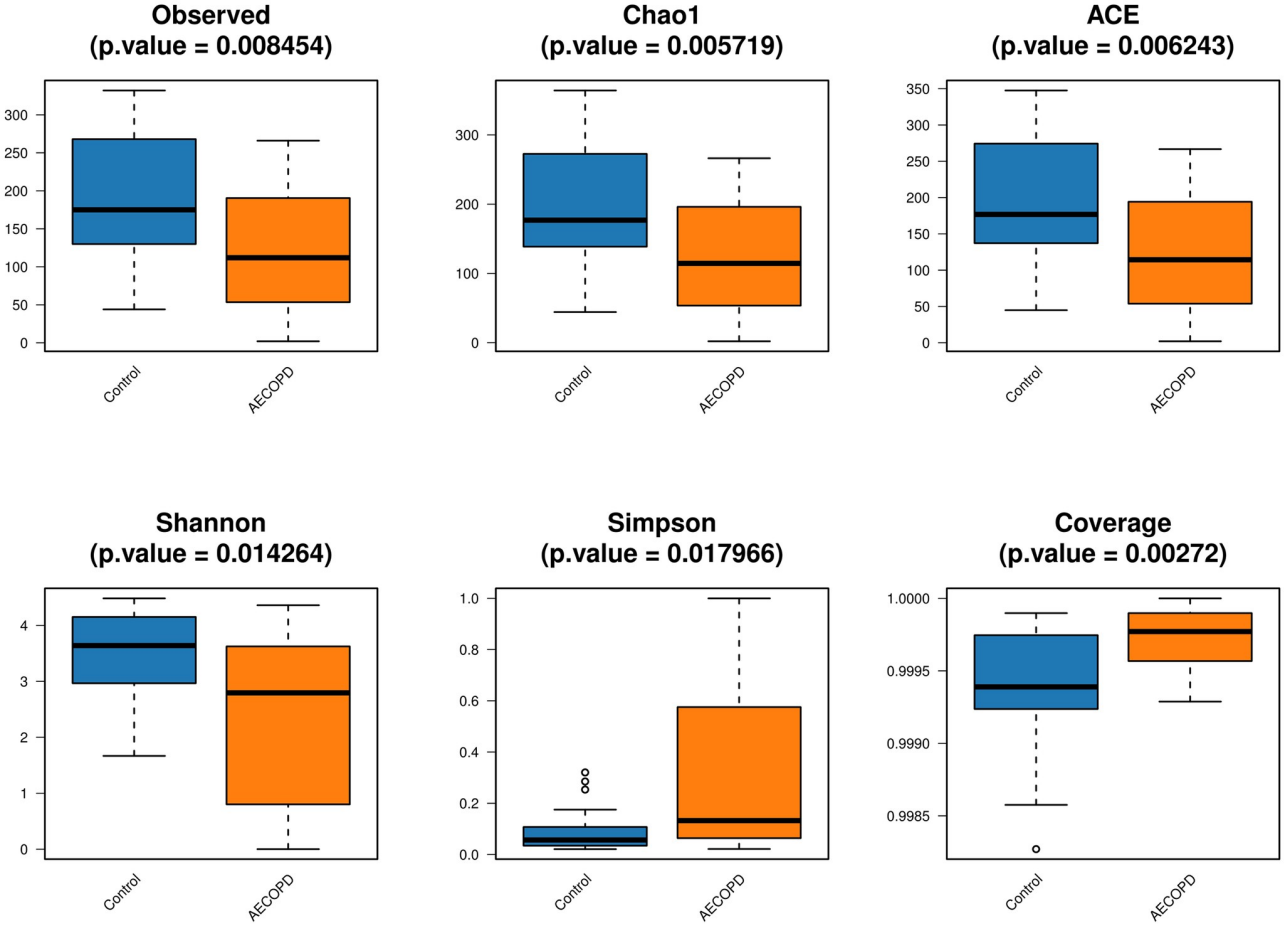
Wilcoxon rank sum test. The Wilcoxon Rank sum test was used to analyze the differences of α -diversity index between AECOPD patients and healthy population.

**Fig 4:**
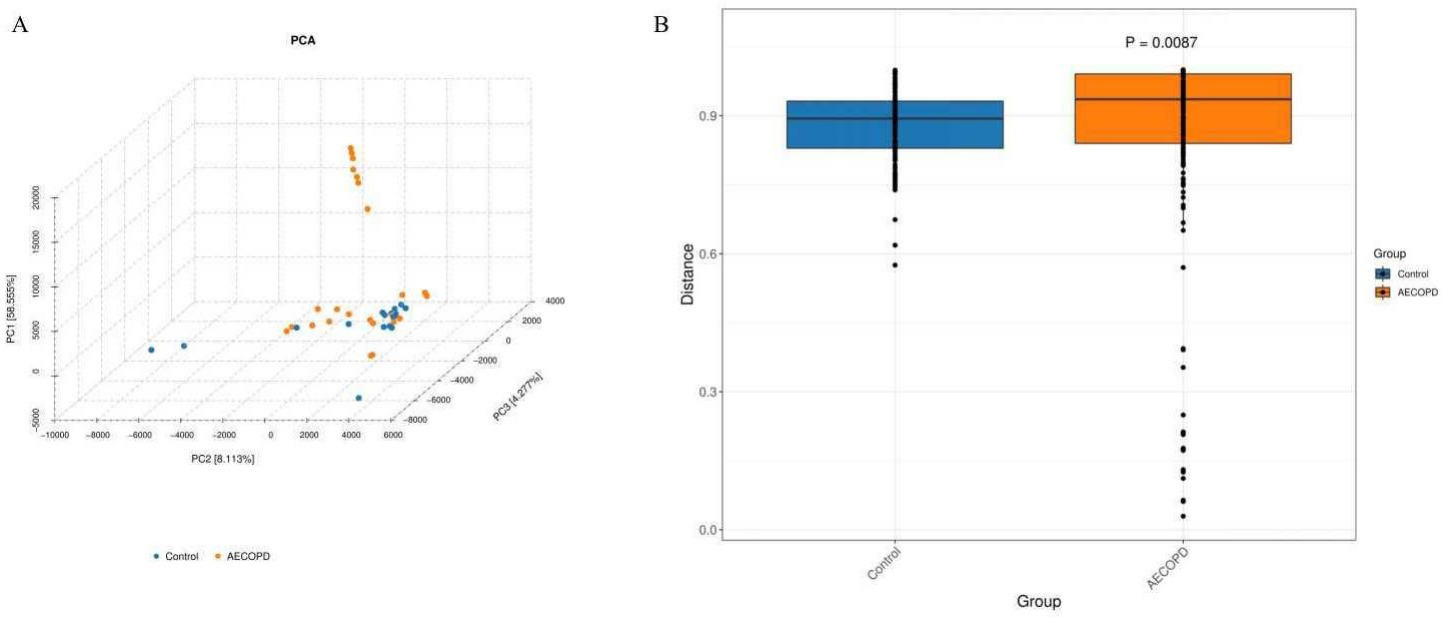
Beta diversity. The beta diversity of the gut microbiome in patients with AECOPD. The principal coordinate analysis (PCA) graph was obtained by calculating the weighted UniFrac distance (A), beta analysis indicated that a remarkable difference was identified between AECOPD group and control group (B).

In total, 2245 OTUs were obtained using Illumina high throughput sequencing analysis. A Venn diagram was used to visualize the number of OTUs and overlaps between the two groups. 629 OTUs were shared between the two groups, with 697 unique OTUs in the AECOPD group and 919 OTUs in the healthy control group (Fig 1). According to the sample number and the number of species OTUs, a species accumulation curve, Shannon curve, rarefaction curve, and a rank-abundance distribution curve of all samples was constructed to evaluate the richness and diversity of the microbiota species (Fig 2). The results showed that the sample number was large enough to reflect adequate species richness and diversity. The Wilcoxon rank sum test suggested that the values of Chao, Observed, ACE, and Shannon in the healthy group were significantly increased compared to the AECOPD patients (all *P*<0.05). However, the Simpson’s diversity index and coverage values were increased in patients with AECOPD. The results indicated that the richness and diversity of gut microbiota decrease during acute episodes of COPD (Fig 3). In addition, a PCA graph was obtained by calculating the weighted UniFrac distance (Fig 4), which displayed the similarity and difference in different environments. The contributions of the principal components PC1, PC2 and PC3 were 58.555%, 8.113% and 4.277%, respectively (*P* = 0.0078, Fig 4). The highest species abundances were selected to demonstrate the distribution differences at the level of phylum, class, order, family, genus, and species, and the dominant phyla were *Firmicutes*, *Bacteroidetes*, and *Proteobacteria* (S1 Fig). Metastat analysis was used to analyze the differences at different levels. The results indicated that there was a significant increase in *Bacteroidetes* at the phylum level in the case group. The *Bacteroidia* class in the healthy control group and the *Bacilli* class in the AECOPD group were abundant. In addition, at the order level, *Bacteroidales* and *Pasteurellales* were abundant in the healthy control group and *Actinomycetales* and *Lactobacillales* were abundant in the AECOPD group. In the level of family, both *Bacteroidaceae* and *Pasteurellaceae* were prevalent in the healthy group, and the *Actinomycetaceae*, *Enterococcaceae*, and *Micrococcaceae* families were frequent in patients with AECOPD. At the genus level, *Bacteroides*, *Ruminococcus*, *Dorea*, *Barnesiella*, *Haemophilus*, and *Veillonella* genera were prevalent in the healthy control group. The abundance of *Bacteriodes_vulgatus* and *uncultured_bacterium_adhufec* 108 was decreased in patients with AECOPD (S2–S7 Figs).

**Fig 5:**
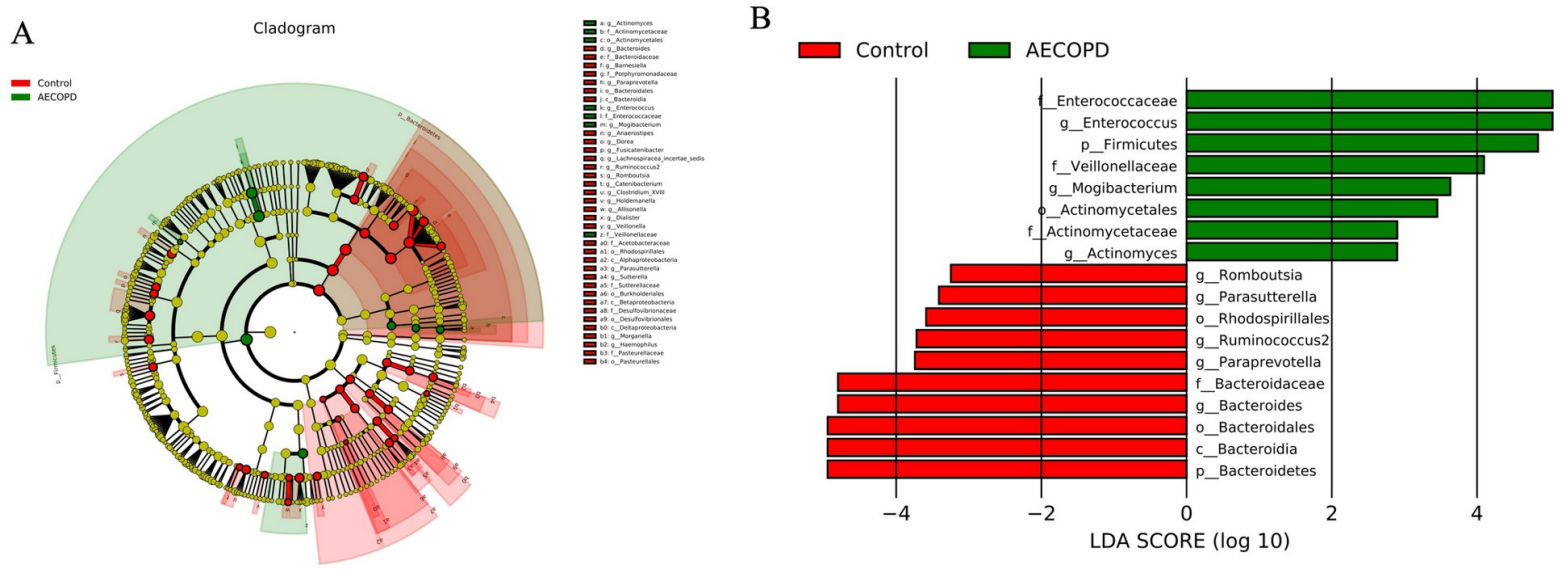
Linear discriminant analysis effect size. LEfSe was used to analyze the differential bacterial abundance (A). The results suggested that the richness of the phylum of *Bacteroidetes*, *g_Paraprevotella*, *g_Ruminococcus2*, *g_Parasutterella*, *o_Rhodospirillales* and *g_Romboutsia* were prevalent in healthy population. However, the abundance of *p_Firmicutes*, *o_Actinomycetales*, *f_Actinomycetadeae*, *g_Actinomyces*, *g_Mogibacterium f_Veillonellaceae*, *f_Enterococcaceae* and *g_Enterococcus* were frequent in AECOPD patients (B).

In addition, LEfSe was used to estimate which bacteria were different in the two groups. The results suggested that the phylum *Bacteroidetes*, and the genera *g_Paraprevotella*, *g_Ruminococcus2*, *g_Parasutterella*, *o_Rhodospirillales*, and *g_Romboutsia* were prevalent in the healthy group. However, the abundance of *p_Firmicutes*, *o_Actinomycetales*, *f_Actinomycetadeae*, *g_Actinomyces*, *g_Mogibacterium f_Veillonellaceae*, *f_Enterococcaceae*, and *g_Enterococcus* were frequent in AECOPD patients (Fig 5).

### Alterations in the function of the gut microbiome

**Fig 6:**
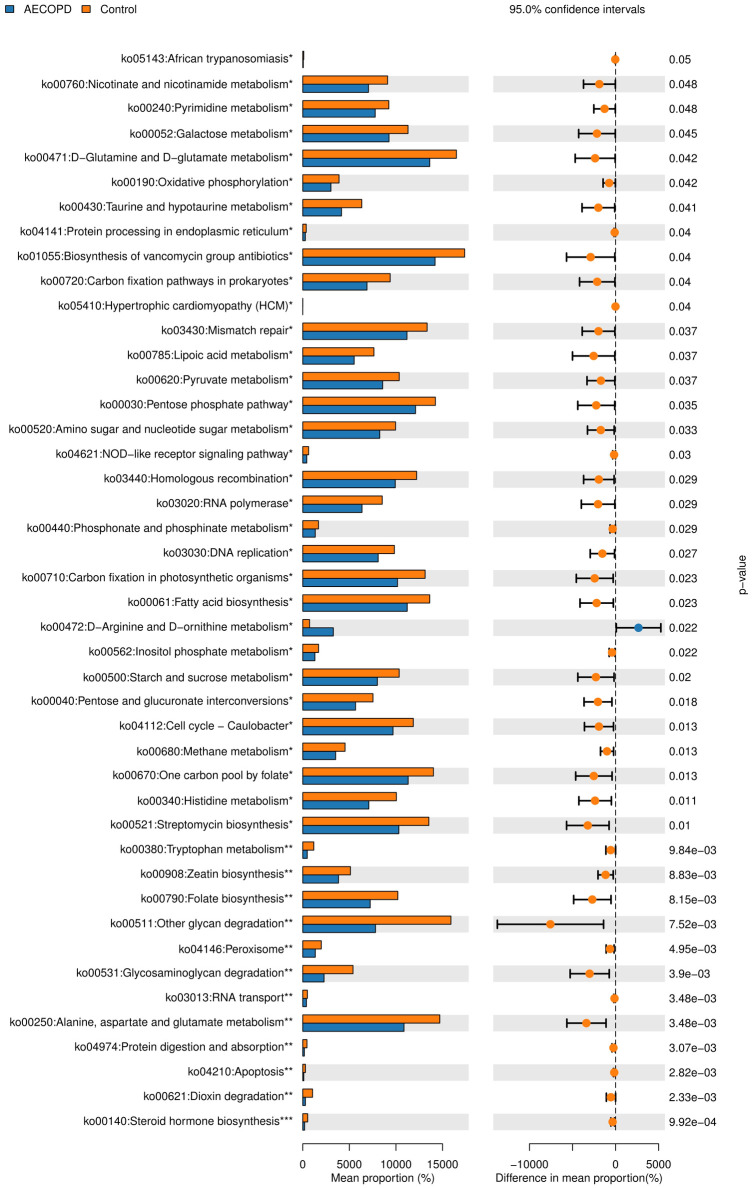
PICRUSt2. Alterations in the function of the gut microbiome was showed by using PICRUSt2 algorithm.

As shown in Fig 6, the functions of the differently expressed gut microbiota genes were analyzed using the PICRUSt2 algorithm, and their biological annotations were referenced from the KEGG database. Compared with healthy group, predicted activity of all metabolism pathways were significantly decreased in the AECOPD group, especially histidine metabolism.

### Alterations of SCFA levels in AECOPD patients

**Fig 7:**
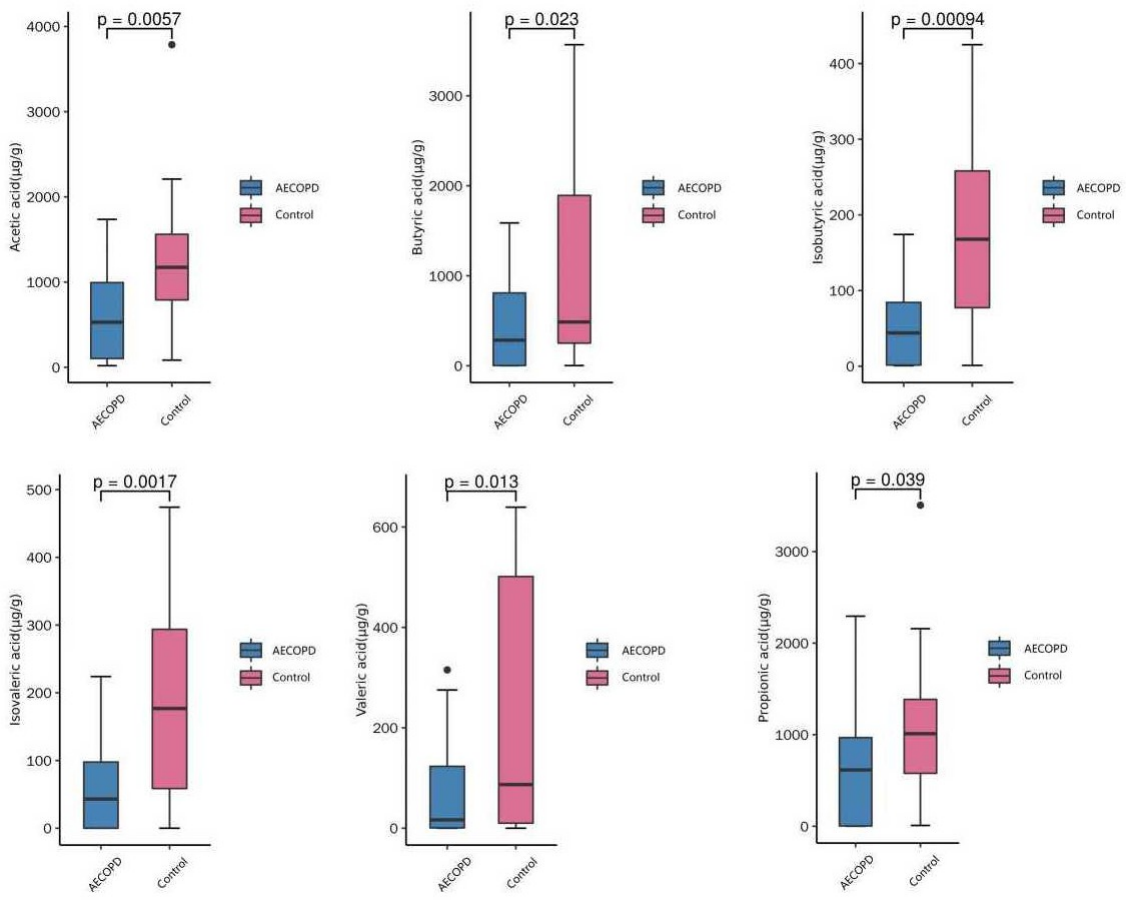
The concentration of SCFA between AECOPD patients and control group.

GC-MS was used to determine the concentration of gut SCFA, including acetic acid, butyric acid, propionic acid, isobutyric acid, valeric acid, and isovaleric acid. The results indicated that SCFA levels were decreased in patients with AECOPD, compared to the healthy group (all *P*<0.05, Fig 7). Furthermore, medium and long chain fatty acids were also analyzed, but there were no differences between the groups (S8 Fig).

### Comparison of inflammation in the healthy and AECOPD groups

In addition to the routine blood test analysis, several inflammatory factors, including TNF-α, IL-1β, IL-6, and IL-10, were measured in the serum obtained from the participants and detected by ELISA. The serum concentration of IL-6 was decreased in AECOPD patients compared to the healthy group (*P* = 0.00). No other parameters were found to be different between the two groups (all *P*>0.05, Table 2).

**Table 2:** The comparison of inflammation factors in AECOPD patients and control groups (pg/mL).

Variables	Control (n = 18)	AECOPD (n = 24)	*P*
TNF-α	151.08±37.53	209.52±170.49	0.170
IL-1β	28.76±8.73	33.81±24.81	0.419
IL-6	15.11±4.29	27.49±12.33	<0.01
IL-10	20.06±6.53	19.66±14.53	0.914

**NOTE** *TNF* Tumor necrosis factor. *IL* Interleukin

## Discussion

The gut microbiota-SCFA-inflammation axis may be an important relationship that connects the gut microbiota and inflammatory respiratory disorders. The present study found that AECOPD patients have decreased abundance and diversity of gut microbiota, and a reduction in luminal SCFA concentrations and serum IL-6 concentration was also observed.

There is a need to explore the difference in structure and abundance of intestinal microflora in healthy and disease states to understand the role of gut microbiota in health and disease. Dysbiosis in gut microflora is evident in patients with COPD [7]. In acute exacerbation of COPD, gut microbiota dysbiosis has been regarded as potential trigger factor [19]. The present study found that the richness and diversity of gut microbiota decreased during acute episodes of COPD, which is in accordance with previous research. Wu *et al*. also found that there was a reduction in the richness and structure of the fecal microbiome during acute episodes of COPD [9]. A study by Bowerman *et al*. revealed that 146 bacterial species in the gut microbiome differ between COPD patients and healthy controls. Several species, including *Streptococcus sp000187445*, *Streptococcus vestibularis* and multiple members of the family *Lachnospiraceae*, were also correlated with reduced lung function [20]. These studies suggest that dysbiosis of gut microbiota maybe had relationship with the acute episode in AECOPD patients.

SCFA can become an inhibitor of histone deacetylase, which promotes the proliferation of T cells and can help restore the imbalance of pro-inflammatory and anti-inflammatory states, forming a link between the gut microbiota and host cells [21, 22]. A rat model of COPD was established by chronic exposure to ambient particulates, which induced reduction of microbial richness and diversity and lower levels of SCFA [7]. Our results are consistent with these previous studies. The present study demonstrated that the concentration of SCFA in the gut lumen was decreased in AECOPD patients compared with healthy controls. We speculate that the structure and abundance of gut microbiota is related to this phenomenon. LEfSe was performed to analyze the difference in gut microbiota between AECOPD patients and healthy controls, which suggested that the order of *Rhodospirillales*, and genus of *Paraprevotella*, *Ruminococcus2*, *Parasutterella*, *Romboutsia* were prevalent in the healthy group, and *g_Mogibacterium*, *f_Enterococcaceae*, and *g_Enterococcus* were freqeuent in AECOPD population. Previous studies show that bacteria in the genera *Paraprevotella*, *Ruminococcus2*, *Parasutterella*, and *Romboutsia* are SCFA-producing species and a reduction in the abundance of these species may explain the lower concentrations of SCFA observed in AECOPD patients [23–25]. Additionally, *f_Enterococcaceae* and *g_Enterococcus* have been associated with a reduction in fecal SCFA levels [26, 27]. *g_Mogibacterium*, which was found to be increased in AECOPD patients in the present study, is associated with impaired gut health and reduced levels of SCFA in the cecum of male Holstein-Friesian calves fed by Sodium-Butyrate [28]. Taken together, these results suggest that dysbiosis is associated with the reduction of SCFA in AECOPD patients, and these species maybe a biomarkers for predict the acute episode of COPD.

As mentioned before, SCFA can play a role in regulation of inflammation by inhibiting histone deacetylase. It has been reported that during the acute episodes of COPD, the reduction of SCFA translates to an increased pro-inflammatory response. The present research indicated that histidine metabolism pathway decreased in AECOPD patients. In addition, we also found that the serum level of IL-6 decreased in AECOPD patients compared with healthy controls. However, other inflammatory factors did not differ between the two groups. The results of our study differ from previously published reports. Aslani *et al*. found levels of IL-6 elevated in COPD patients compared to the control group [29]. In addition, Huang *et al*. reported that in AECOPD, inflammatory responses were increased, and patients with high serum IL-6 experienced more frequent exacerbations of COPD [30]. Obling *et al*. research suggested that patients with COPD had increased of IL-1β in serum [16]. This disparity between the results of the present study and previous studies may be explained by the fact that dramatic inflammatory reactions only occur in the lungs, and no excessive of inflammatory factors released into peripheral blood in current study.

The current study has several limitations. First, this study did not look at local airway inflammation because of the difficulty in obtaining samples. Another significant limitation of our study is the small sample size; only 24 patients with AECOPD and 18 healthy volunteers were included in the analysis. Finally, unfortunately, stable COPD patients were not included in the study. In the future, we would like to increase the sample size and address the above limitations. The study of changes in the gut microbiota of AECOPD will help us further understand the role of the gut microbiota in COPD and AECOPD, so as to better understand the pathogenesis of COPD to AECOPD.

In conclusion, the diversity and abundance of gut microbiota was decreased in patients with AECOPD, which had associated with the reduction fecal SCFA concentrations. In addition, a reduction of IL-6 concentration was also identified.

## Supporting information

S1 Fig The relative abundance of species was demonstrated at level of level (P: Phylum; C: Class; O: Order, F: Family, G: Genus, S: Species). (TIF)

S2 Fig The metastat analysis was used to analyze the differences at phylum levels. (TIF)

S3 Fig The metastat analysis was used to analyze the differences at class of levels. (TIF)

S4 Fig The metastat analysis was used to analyze the differences at order of levels. (TIF)

S5 Fig The metastat analysis was used to analyze the differences at family of levels. (TIF)

S6 Fig The metastat analysis was used to analyze the differences at genus of levels. (TIF)

S7 Fig The metastat analysis was used to analyze the differences at species of levels. (TIF)

S8 Fig The concentration of medium and long chain fatty acids between AECOPD patients and control. (TIF)

S1 File (7Z)

S2 File (7Z)

S3 File (XLSX)

S4 File (XLSX)
