# Supplementary figures and images for: The gut microbiota-SCFA-inflammation axis in patients with AECOPD
Source: PLoS One. 2025 Jan 9;20(1):e0312606. doi: 10.1371/journal.pone.0312606 (PMC11717293; doi:10.1371/journal.pone.0312606)

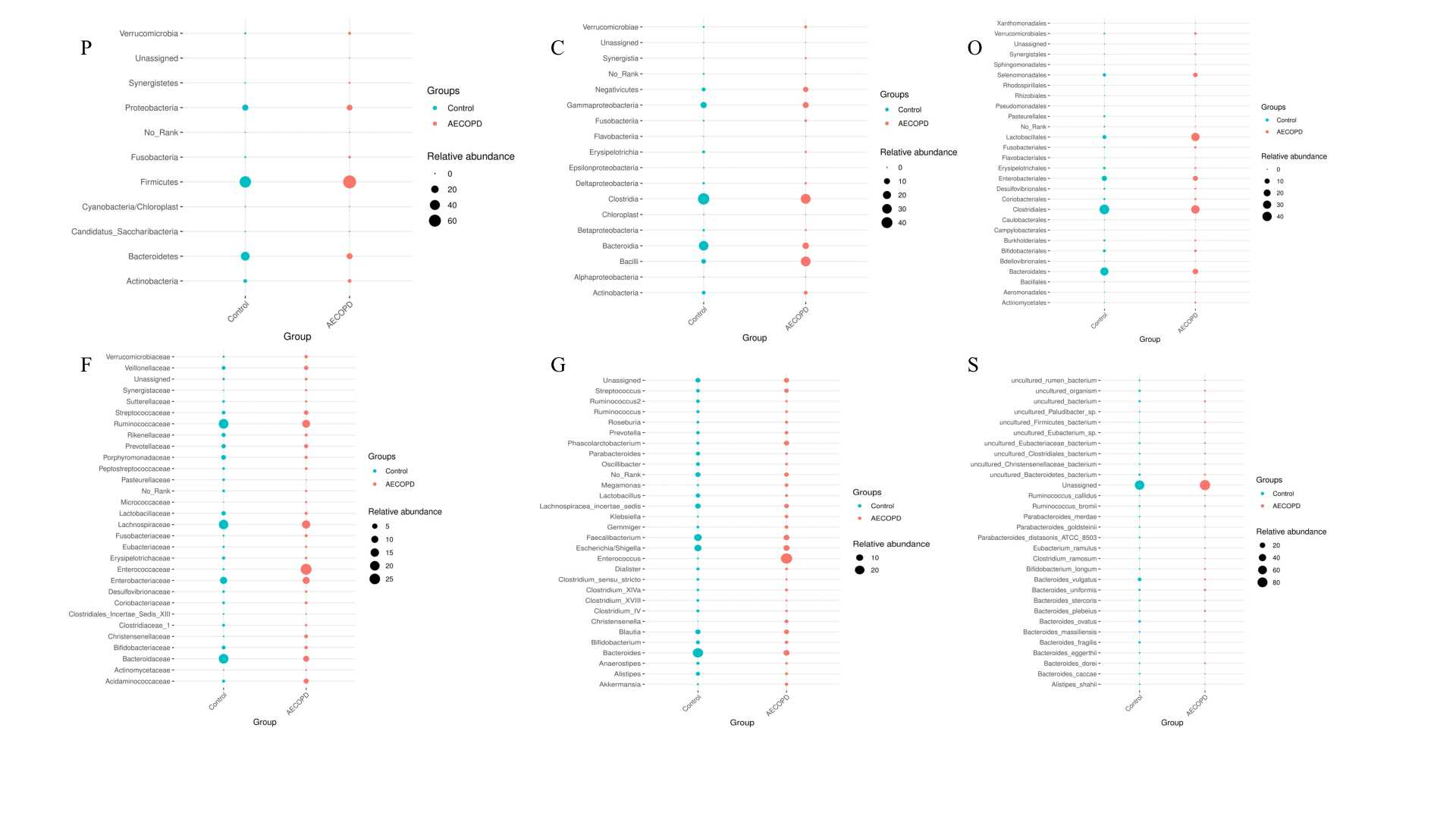

Supplement: S1 Fig — (TIF) [file pone.0312606.s001.tif]

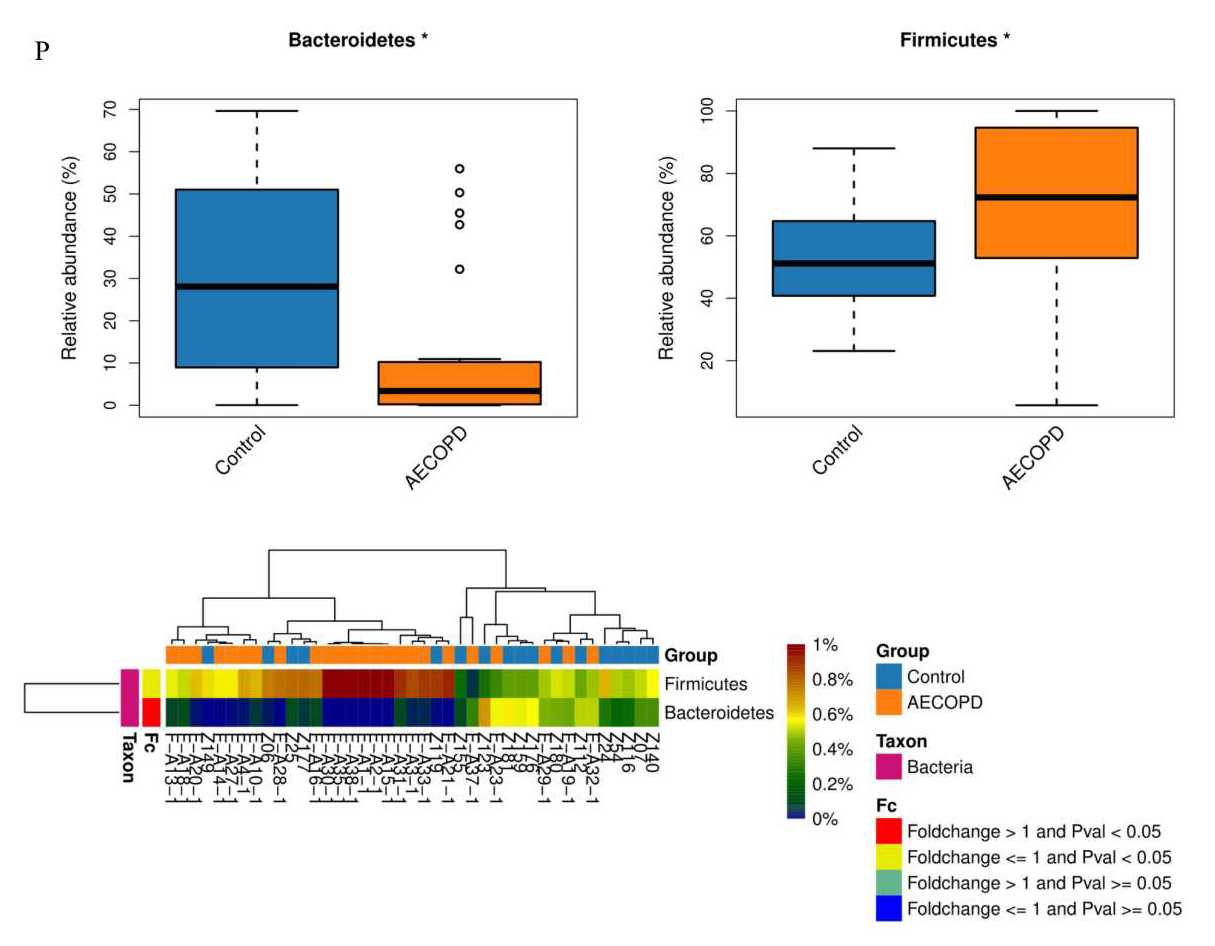

Supplement: S2 Fig — (TIF) [file pone.0312606.s002.tif]

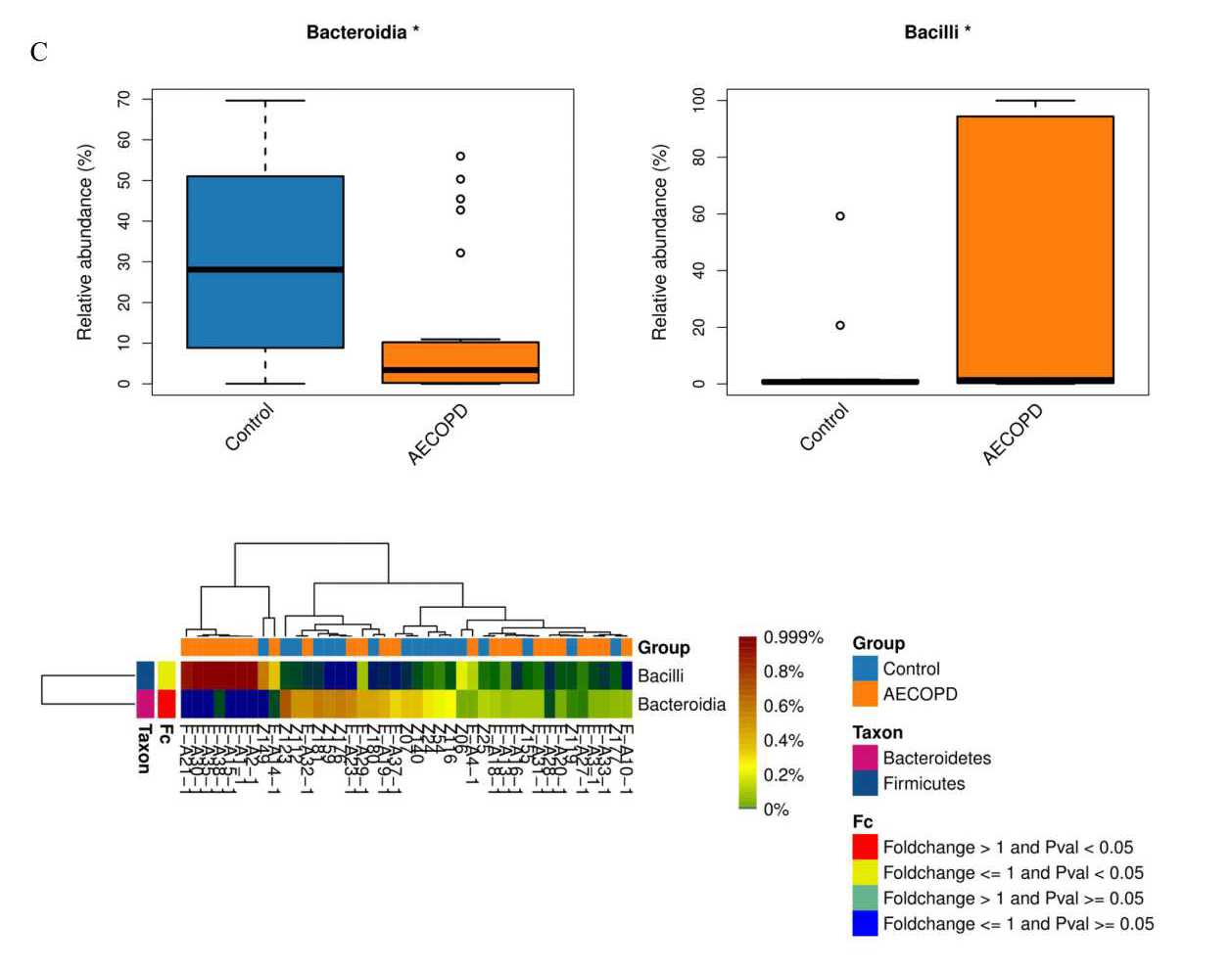

Supplement: S3 Fig — (TIF) [file pone.0312606.s003.tif]

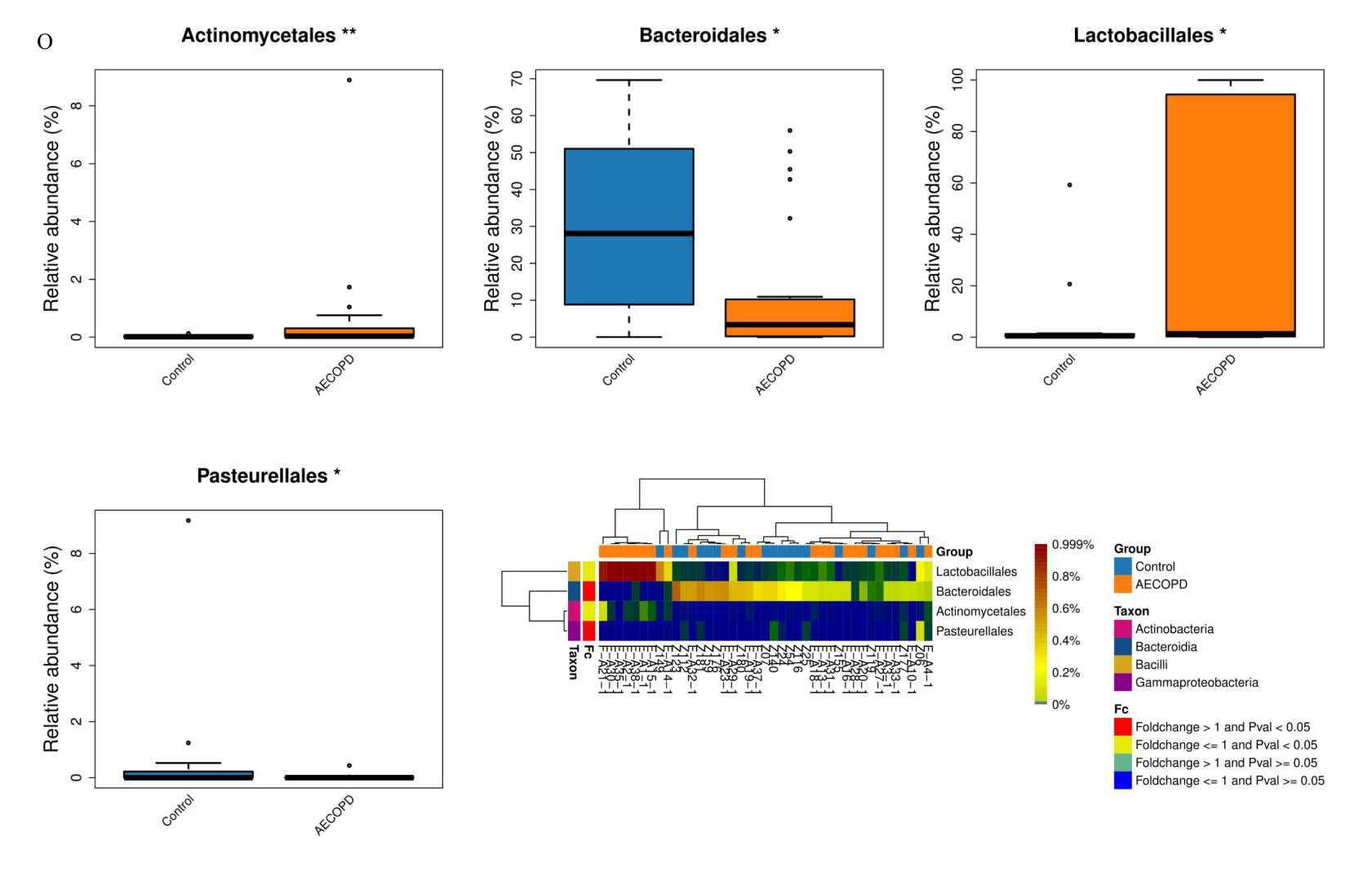

Supplement: S4 Fig — (TIF) [file pone.0312606.s004.tif]

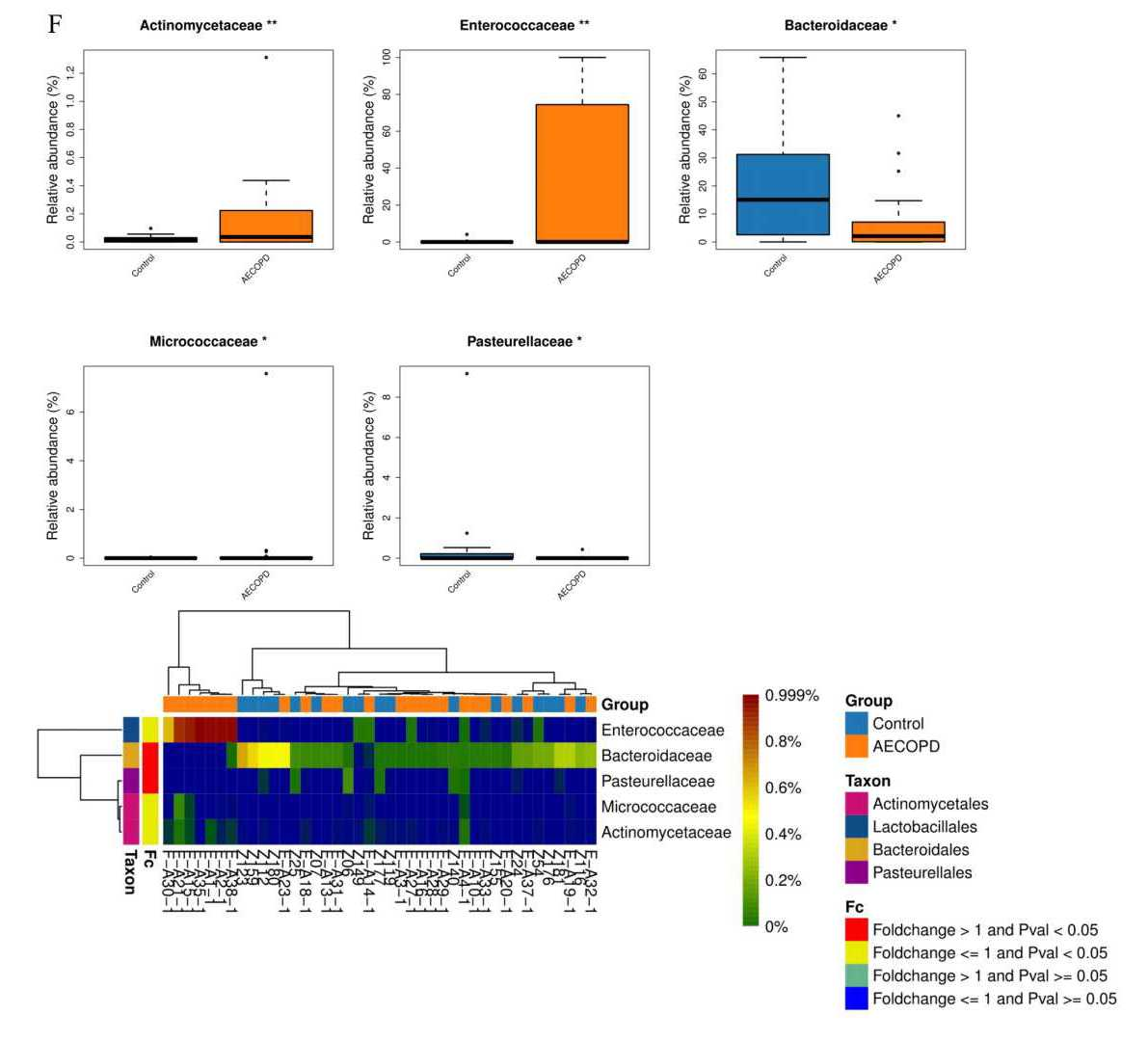

Supplement: S5 Fig — (TIF) [file pone.0312606.s005.tif]

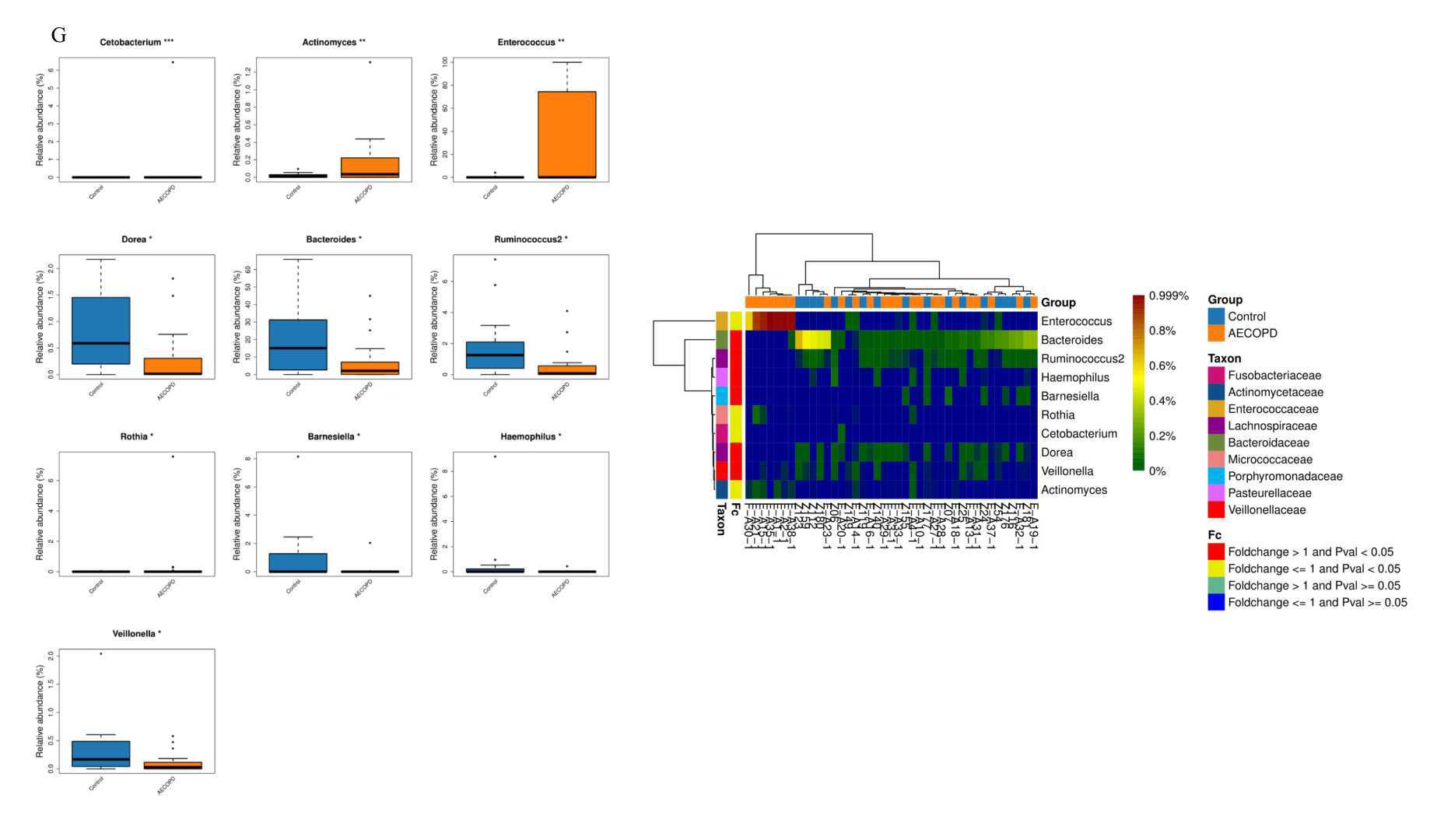

Supplement: S6 Fig — (TIF) [file pone.0312606.s006.tif]

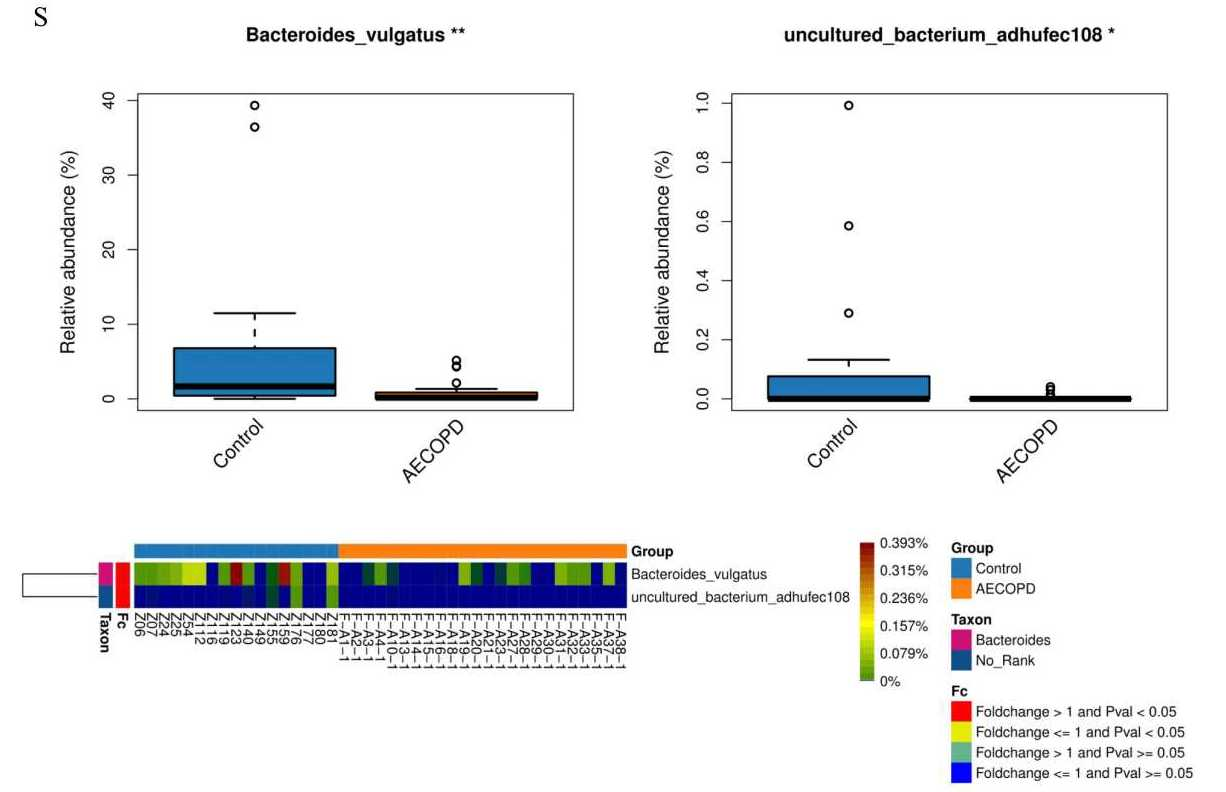

Supplement: S7 Fig — (TIF) [file pone.0312606.s007.tif]

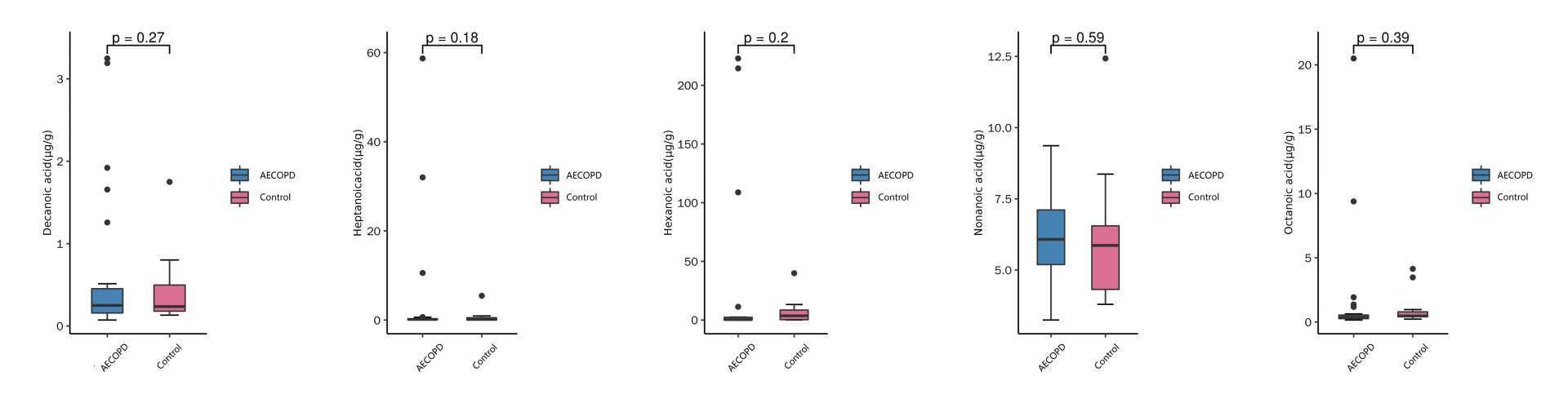

Supplement: S8 Fig — (TIF) [file pone.0312606.s008.tif]
